# Tackling (Childhood) Obesity through a Voluntary Food Reformulation Policy: A Repeated Cross-Sectional Study Investigating Nutritional Changes in the Out-of-Home Sector

**DOI:** 10.3390/nu15143149

**Published:** 2023-07-14

**Authors:** Tammy Pepper, Kathryn H. Hart, Charo E. Hodgkins

**Affiliations:** 1 Department of Nutritional Sciences, Faculty of Health & Medical Sciences, University of Surrey, Guildford GU2 7XH, UK; 2 School of Psychology, Faculty of Health & Medical Sciences, University of Surrey, Guildford GU2 7XH, UK

**Keywords:** obesity prevention, nutrition, reformulation, sugar reduction, food environment, out-of-home, chain restaurants, policy evaluation

## Abstract

The Childhood Obesity Plan aimed to reduce sugar and energy in foods through a voluntary sugar-reduction programme. Our primary objective was to determine whether this implementation strategy had been successful, focusing on the out-of-home sector. We used a repeated cross-sectional design to evaluate nutritional changes in desserts served by leading chain restaurants. We extracted nutrition information from online menus in autumn/winter 2020, for comparison with baseline (2017) and interim (2018) values extracted from third-party datasets. We assessed compliance with the 20% sugar-reduction target and category-specific energy targets by product category and for pooled desserts. Overall, sugar/portion and energy/portion decreased by 11% and 4%, respectively. Policy targets were achieved in one of five categories (ice-cream: −38% sugar, *p* < 0.001; −30% energy, *p* < 0.001). Secondary outcomes were analysed for subgroups with the necessary data. Few chains significantly reduced sugar and/or energy across their dessert range. Energy/portion was positively associated with portion weight and sugar/portion but not with sugar/100 g. More than half of adults’ desserts contained excessive sugar and/or saturated fat compared with dietary guidelines. Children’s desserts less frequently exceeded guidelines. These results demonstrate that voluntary measures can drive substantial change when technical, commercial, and operational barriers can be overcome.

## 1. Introduction

Excess weight affects 30% of children in England [1] and 18% globally [2,3]. Short-term consequences include increased risk of respiratory, musculoskeletal, and psycho-social issues [4,5]. In the longer term, it is associated with earlier onset of cardiometabolic diseases [5,6,7], predisposes to obesity in adolescence [8,9] and adulthood [10], and increases risk of premature mortality in later life [11]. Therefore, preventing childhood obesity has been highlighted as a public health priority with potential for improving health throughout the life-course [12,13,14], and has been an explicit target in UK health policies since 1991 [15].

Failure to stem the relentless increase, especially for the most deprived, has been attributed to an overemphasis on policies focusing on individual responsibility, with insufficient recognition of the wider determinants of health that constrain lifestyle choices [16,17,18,19,20]. Indeed, the food environment has a pivotal role [21,22,23] whereby increased food abundance [24], reliance on energy-dense processed food [25,26], normalization of large portion sizes [27,28], and proliferation of fast-food outlets/home-delivery platforms [29,30] contribute to excess consumption by susceptible individuals.

To counter these obesogenic stimuli, public health bodies advocate reformulation and reducing portion size as high-priority actions [12,13,31]. The intention is to improve the nutritional profile of habitually consumed foods through gradual, silent reformulation, thereby helping consumers improve their dietary composition without conscious behaviour change [32]. Sugar reduction has been given particular emphasis as the evidence suggests a causal relationship between free sugars and bodyweight in free-living individuals, mediated by energy intake [33,34,35,36,37]. Accordingly, the commitment to reduce sugar in foods and beverages formed a central pillar of the UK Government’s Childhood Obesity Plan (COP). It was implemented through a voluntary reformulation programme, with the underlying threat of stronger levers if targets were not achieved, as recommended by the House of Commons Health Committee [38,39].

A structured sugar-reduction programme (SRP) [40], claimed to be a global first [41], was developed and administered by Public Health England (PHE), an executive agency of the Department of Health and Social Care, and, subsequently, transferred to the Office for Health Improvement and Disparities (OHID). Key success factors were identified as strong central leadership, mandating action throughout the food industry to create a level playing field, setting reformulation targets for a wide range of foods to increase impact, and effective and transparent monitoring [40,42].

The SRP encompassed food categories making the greatest contribution to free sugars in children’s diets and applied to both retail and out-of-home (OOH) products. The goal was 5% reduction by August 2017 and 20% reduction by 2020, compared with 2015 averages, to be achieved by reformulation, reducing portion size, or shifting sales to lower-sugar products. Category-specific targets were also set for energy per portion [43].

From the outset, some public health experts criticized the lack of sanctions [44,45] while others questioned the single nutrient focus of the programme and the extent to which it would reduce energy content in foods [46]. The latter is particularly pertinent for an obesity-prevention initiative and warrants validation. However, modelling studies did not make provision for the energy (or other nutrients of concern) contributed by replacement ingredients [47]. At a time when the UK has been chosen to lead food reformulation initiatives for the WHO (World Health Organization) European region [48], and, in view of the SRP’s controversial reliance on industry co-operation, it is important to examine whether the voluntary approach adopted was sufficient to drive action and also to investigate the broader nutritional impact.

In the OHID’s final report [49], the 20% sugar-reduction target was not achieved in any food category, although greater changes were observed for many milk-based drinks (MBD) subject to different deadlines. Fewer than half of the top 20 brands in each food category reduced sugar by more than 2%. Undesirably, saturated fat increased alongside sugar reduction for 14% of the top-selling brands. Nevertheless, OHID concludes “…a voluntary sugar reduction and product reformulation programme can deliver progress, change and innovation”, evidenced by progress in retail breakfast cereals (−14.9%), yogurts (−13.5%), and MBD (−29.7%).

This investigation focuses on OOH foods, which account for 18% of meals [40] as they are covered in much less detail and the interpretation of results is complicated by successive changes in key parameters. Notably, the OOH baseline was changed to 2017; reporting metrics were changed from sales-weighted to simple averages, and sugar and energy were analysed for different sets of products (the sample size was substantially lower for sugar). Furthermore, saturated fat was not monitored in OOH foods.

At the time of writing, there is only one peer-reviewed paper with longitudinal data for restaurants in the UK [50]. However, it is not possible to identify sugar reduction motivated by SRP. Crucially, the main analyses combined data for all menu items, including soft drinks (subject to taxation [51]) and savoury foods (outside the scope of SRP). Additionally, although stratified analyses reported changes for desserts, different types of dessert were not separately analysed and baked goods were not included. Moreover, 2020 values were compared with 2018 values rather than 2017 values (OOH baseline).

Our primary objective was to review progress towards SRP targets in large restaurant chains. Our results complement the official follow-up by tracking changes in sugar on a per portion basis rather than per 100 g (the designated reporting metric) which cannot capture sugar reduction achieved through portion control. The latter is particularly relevant in the OOH sector where portion sizes are larger and determined by the operator rather than the consumer.

We aim to extend the literature by (i) analysing nutritional changes by SRP category from baseline to end-point (2017–2020), (ii) monitoring sugar and energy reduction by brand, (iii) investigating correlations between sugar and energy content (the underlying premise for SRP), and (iv) comparing nutritional values with dietary guidelines for adults’ and children’s menus. Finally, we will discuss implications for future reformulation programmes.

## 2. Materials and Methods

### 2.1. Design and Setting

We used a repeated cross-sectional design to examine 2017–2020 changes in the nutritional profile of desserts served by major restaurant chains in the UK. We sourced nutritional data from online menus in Q4 2020 for comparison with baseline (2017) values and interim (2018) values extracted from third-party datasets.

#### 2.1.1. Restaurant Inclusion Criteria

We included: (i) full-service restaurants (FSR), comprising pub and casual dining restaurants with table service; (ii) quick-service restaurants (QSR) (including both fast-food and fast–casual restaurants) characterized by simple menus, value, or intermediate pricing, with a takeaway focus and basic on-site dining; and (iii) coffee-shops, cafés, and bakery-led chains (CCB). Other channels constitute < 30% of the OOH market [52,53,54,55,56,57,58,59,60] and nutritional data was not accessible online. Chains with at least 20 outlets, the threshold triggering nutritional labelling in North America [61,62], serving relevant products and providing nutritional information online, were deemed eligible for inclusion.

**Figure A1:**
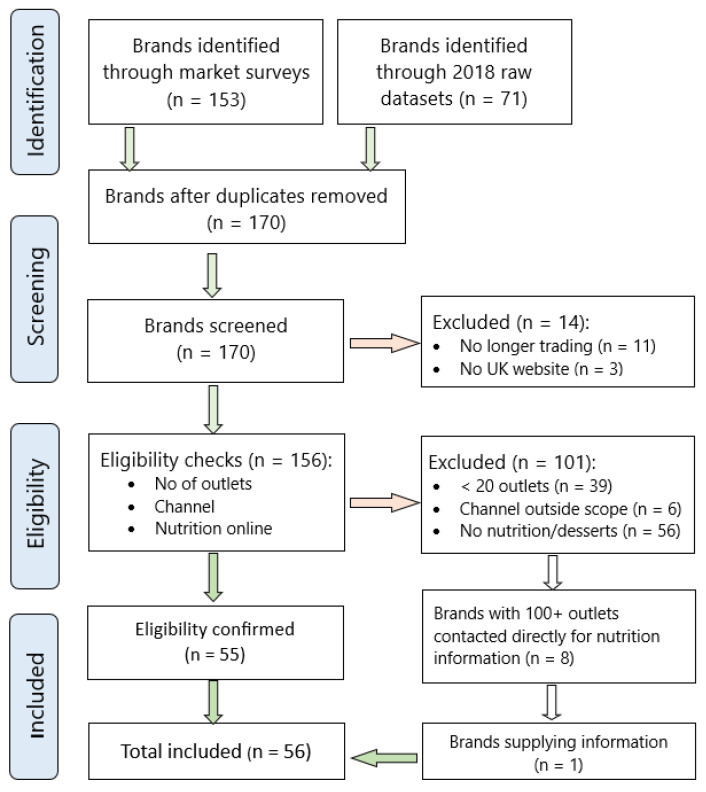
Summary of restaurant sampling procedure for 2020, flowchart adapted from PRISMA [118].

The sampling procedure for 2020 is shown schematically in Appendix A Figure A1. We combined leading restaurant chains identified from market surveys ranking brands by popularity [63,64,65], outlets [66,67,68], or users [69] with brands featuring in the 2018 datasets. After removal of duplicates, we screened 170 brands for eligibility by checking company websites to confirm the type of restaurant, number of outlets, and availability of nutritional information for relevant products. Brands with 100+ outlets were contacted directly for nutrition information if it was not available online. The final sample included 56 brands.

#### 2.1.2. Product Inclusion Criteria

We included biscuits, cakes, ice-cream, morning goods, and puddings (collectively termed desserts in this report). These categories are the most relevant to the OOH sector and align with OHID analysis.

### 2.2. Data Collection and Extraction

We collected original data from company websites between September and December 2020, immediately following the August 2020 due date for achieving sugar-reduction targets [70]. Online menus (main menu/dessert menu and children’s menu) and associated nutritional information were downloaded. Nutritional values and portion weights for items within the project scope were manually transcribed into Excel in the format provided (per portion and/or per 100 g). Where portion weights were provided, they were used to compute sugar per 100 g and vice versa. Where portion size was unclear (e.g., ice-cream quoted per scoop, but number of scoops not stated) only the default values were extracted. Similarly, nutritional information for items intended for sharing was documented on a per portion basis, but excluded if the number of portions was vague (e.g., serves 6–8). Products were categorized according to the descriptions in PHE’s technical report (Supplementary File S1) with additional coding for restaurant channel, brand, and menu type (adults’ or children’s).

Customizable toppings (e.g., cream, custard, ice-cream, and confectionery sprinkles) were an integral part of many desserts. For some products, nutritional values referred to the complete dessert. In others, nutrition for toppings was quoted separately. Since the energy target includes an additional allowance for toppings, and to ensure consistency, nutrition for the composite dessert was calculated from the sum of the constituents for all combinations explicitly listed on the menu (e.g., apple pie with custard and apple pie with ice-cream).

For prior years, relevant entries were extracted from third-party datasets. PHE supplied spreadsheets containing unpublished raw data for the years ending August 2017 and August 2018 [71,72] used in the preparation of their interim reports [73,74,75]. The original sources were restaurant websites and information provided by companies [43]. Additional raw data for 2018 (originally obtained from restaurant websites) were available as a supplement to a previous cross-sectional study [76].

Both 2018 files included restaurant names, product coding, and detailed product names/descriptions which were used to identify chains/products fulfilling the inclusion criteria. After extracting relevant entries and eliminating duplicates, coding was added as described above. Data extraction from the 2017 file was based solely on product coding, as restaurant and product names were withheld.

### 2.3. Data Cleaning and Statistics

Internal validity of the data was checked by comparing energy calculated from macronutrients with the quoted energy (kcal/portion). Items with incomplete macronutrient data or discrepancies greater than ±20% were excluded as internal consistency could not be confirmed. Extreme outliers were identified and checked.

Data were analysed in SPSS version 28 [77]. Normality was examined using the Shapiro–Wilk test and by inspecting histograms and Q–Q plots [78]. Non-parametric statistical methods were employed as distributions for energy and macronutrients per portion were positively skewed. All statistical tests were two-tailed using *p* < 0.05 as the threshold for significance. 

#### 2.3.1. Primary Outcome: Progress towards PHE Targets (2017–2020)

Changes in median sugar per portion were calculated by product category and for pooled desserts. Achievement/non-achievement of the sugar-reduction target was defined by 20% reduction in median sugar per portion compared with baseline. Kruskal–Wallis tests followed by Mann–Whitney *U*-tests with Bonferroni correction for multiple comparisons were performed to investigate significant changes between years. Changes in median energy and fat were also analysed to assess broader nutritional impact.

PHE defined the energy targets as absolute values for each category, not percentage reductions. Therefore, achievement/non-achievement was determined by frequencies below the specified thresholds, i.e., ≥50% below target average and 100% of products below target maximum. The chi-squared test was used to investigate time trends.

#### 2.3.2. Sub-Group Analyses (Secondary Outcomes)

As baseline data were anonymized, they could not be included in analyses stratified by brand or menu. Mann–Whitney *U*-tests were used to assess 2018–2020 changes for brands providing nutritional information in both years and to compare nutritional values for adults’ and children’s products by category and effect sizes were calculated [79].

To establish the key determinants of sugar and energy per portion, we investigated associations between different nutritional parameters (sugar, energy, fat, and portion weight). Pearson correlations (stratified by menu) were computed for the subset of products (approximately, 32% of items) with the requisite data. Additionally, we controlled for portion weight using partial correlations.

To identify future reformulation priorities, sugar, energy, fat, and saturated fat in 2020 were compared to the daily reference intakes (RI) stipulated in EU Regulation 1169/2011 on the provision of food information to consumers [80] and guidance that main meals and snacks should provide 30% and 20%, respectively, of the daily amount [81]. Accordingly, thresholds of 30% (full meal allocation) and 50% of the daily amount were used as cut-offs designating ‘excessive’ and ‘extreme’ levels for a dessert or snack. SRP tracks total rather than free sugars; 30% RI total sugar (27 g) is slightly lower than the guideline maximum of 30 g/day of free sugars [40]. For children’s menus, thresholds were calculated pro rata based on the estimated average energy requirement for children aged 2–12 years [82].

## 3. Results

### 3.1. Sample Composition

The sample included 78 restaurant chains of which 71 provided nutritional data in 2018, 56 provided data in 2020, and 48 brands provided data in both years (Supplementary File S2). Brand names were not included in the 2017 dataset, so the number of chains is unknown. Cakes, ice-cream, and puddings were the largest categories (Table 1).

### 3.2. Primary Outcome—Progress towards PHE Targets 

Descriptive statistics for sugar, energy, and fat by category and by year are summarized as boxplots in Supplementary File S3. Saturated fat is also included for 2018 and 2020, but was unavailable for 2017. It did not change significantly in this period.

#### 3.2.1. Sugar Reduction: Percentage Change from Baseline

**Figure 1:**
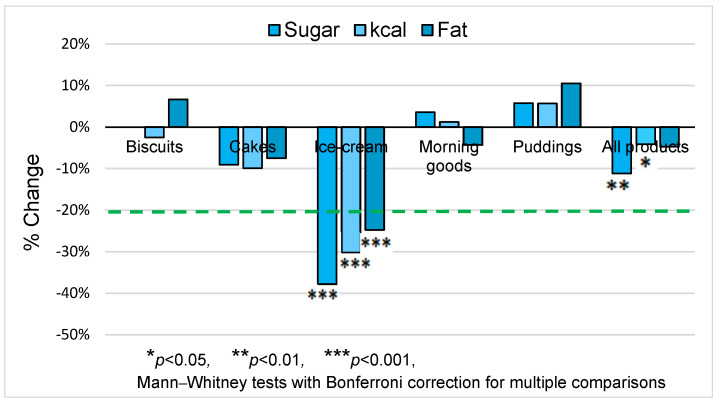
Percentage change in nutritional values per portion (2020 vs. 2017) by product category. Green dashed line represents the sugar-reduction target.

The percentage change in median sugar, energy, and fat per portion between baseline and end-point is shown in Figure 1. Overall, sugar/portion decreased from 30.5 g to 27.1 g (−11%, *p* = 0.001) and energy/portion from 367 kcal to 352 kcal (−4%, *p* = 0.023); fat/portion did not change significantly. Ice-cream was the only category achieving and exceeding the 20% sugar-reduction target. Sugar decreased from 40.5 g to 25.2 g (−38%, *p* < 0.001), energy decreased from 322 kcal to 225 kcal (−30%, *p* < 0.001), and fat decreased from 11.2 g to 8.4 g (−25%, *p* < 0.001). In cakes, the downward trends for sugar (−9%) and energy (−10%) approached significance (*p* = 0.054 and *p* = 0.065, respectively). In other categories, changes were small and not statistically significant.

#### 3.2.2. Energy Reduction: Proportions Meeting Category Thresholds

**Figure 2:**
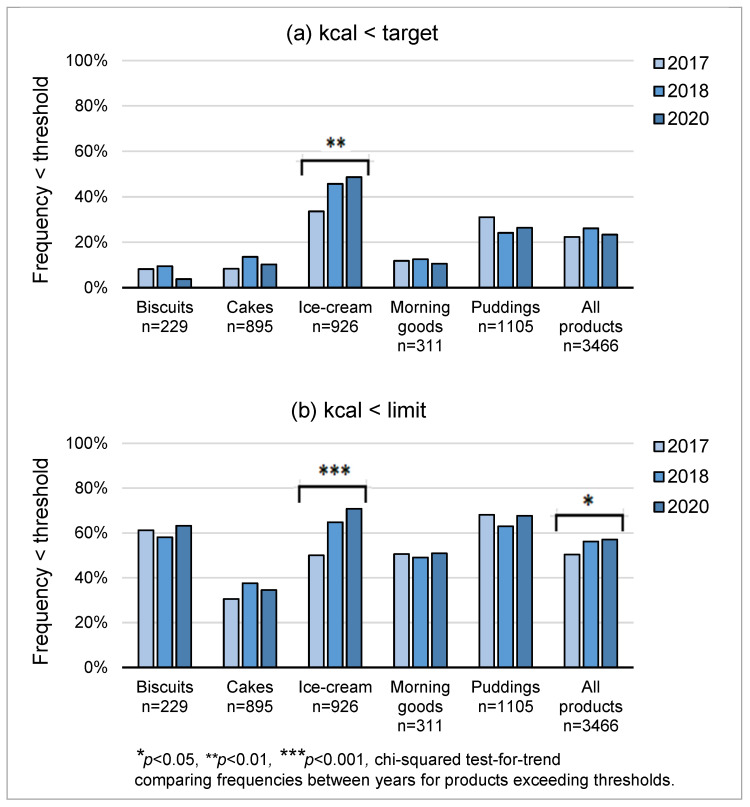
Proportions of products meeting PHE targets for energy per portion by year, (**a**) average values, and (**b**) maximum values. Target averages: biscuits 100 kcal, cakes 220 kcal, morning goods 220 kcal, ice-cream 220 kcal, and puddings 325 kcal. Maximum values: biscuits 325 kcal, cakes 325 kcal, morning goods 325 kcal, ice-cream 325 kcal, and puddings 550 kcal with additions.

Figure 2a,b show the frequencies of products meeting PHE targets for average and maximum kcal/portion, respectively. Ice-cream was the only category to show significant improvement. The frequency below the target average increased from 34% to 49% (*p* = 0.002), i.e., target virtually achieved. The proportion of ice-creams below the upper limit increased from 50% to 70% (*p* < 0.001), contributing to an increase from 50% to 57% in pooled desserts (*p* < 0.05), i.e., target not met.

### 3.3. Secondary Outcomes

#### 3.3.1. Business Compliance

**Figure 3:**
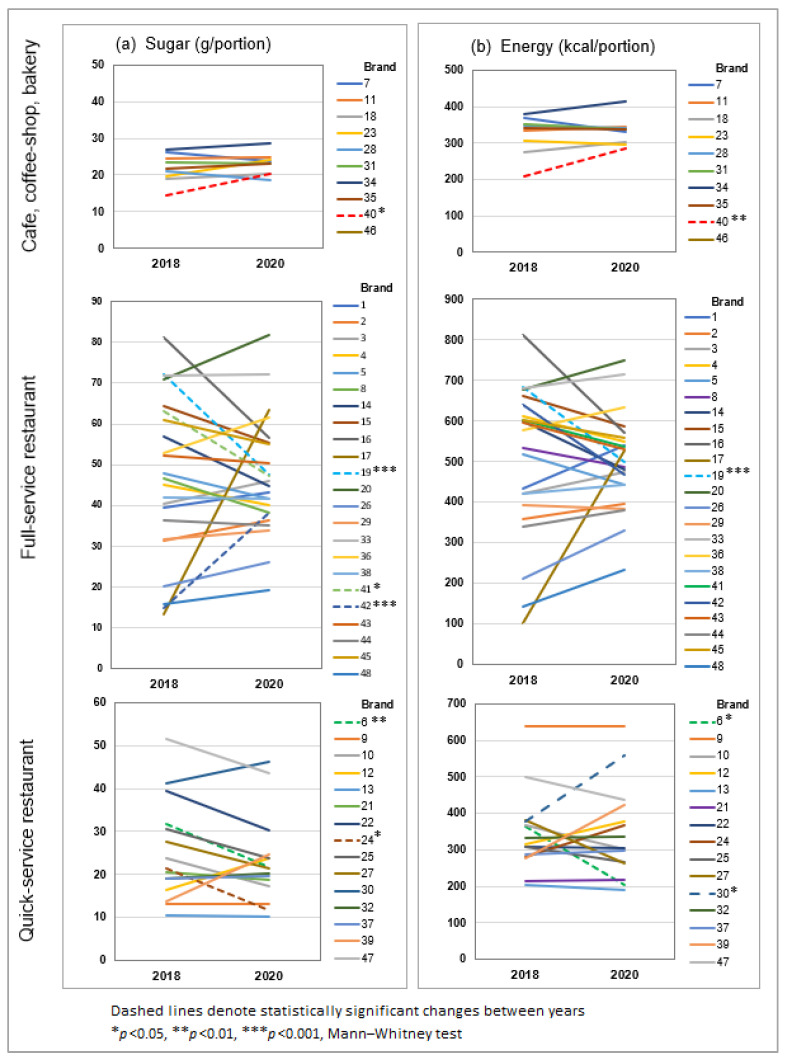
Nutritional changes by brand: (**a**) sugar per portion, and (**b**) energy per portion. Values represent averages for all desserts on menu.

Figure 3 illustrates the variation in sugar and energy content by brand, organized by type of restaurant. Up to four-fold differences in average values were observed within the same channel, including large differences between similar chains. The extremes represented entirely different styles of food, e.g., Japanese and pizza chains within QSR. Only 4 of 48 brands significantly decreased sugar in desserts (reductions of 20–39%, Figure 3a), of which half also decreased energy (18–63% reduction, Figure 3b). Others implemented too few changes to have significant impact. Adverse changes were implemented by three brands, whereby either sugar, energy, or both increased.

#### 3.3.2. Impact of Nutrients and Portion Weight on Sugar and Energy per Portion

Data for portion weight and sugar/100 g were available for approximately one-third of products, of which 94% of items were adults’ desserts. Descriptive statistics for this subgroup are summarized in Supplementary File S4. Per portion values are somewhat lower than for the entire sample population as restaurants in the CCB and QSR sectors provided information on a per 100 g basis more frequently than FSR where desserts were larger and more elaborate.

**Figure 4:**
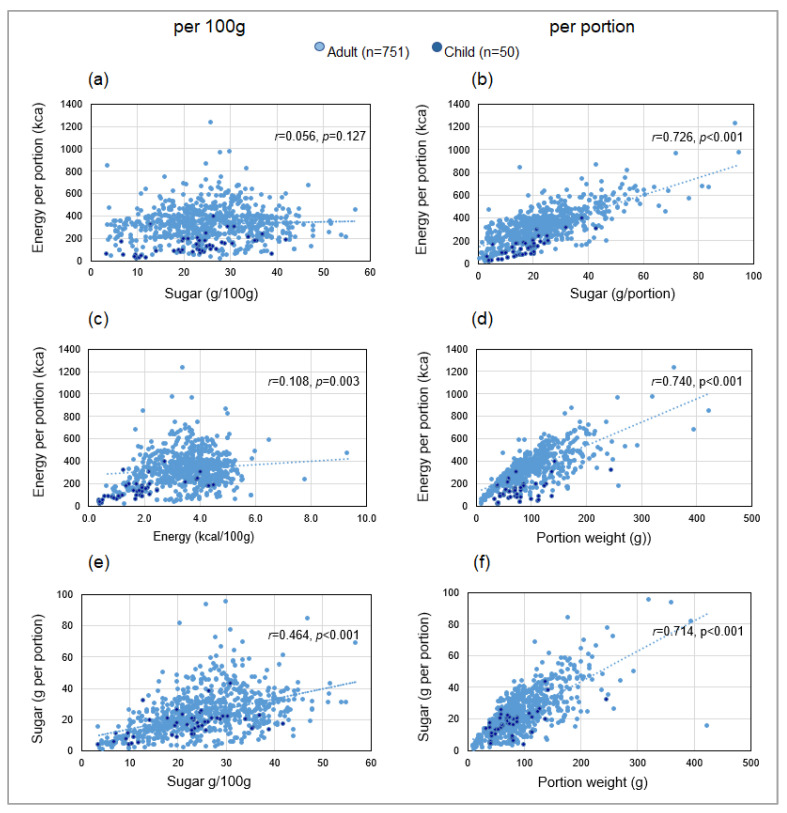
Scatterplots showing correlations between sugar, energy, and portion weight in adults’ and children’s desserts: (**a**) sugar/100 g vs. energy per portion, (**b**) sugar/portion vs. energy/portion, (**c**) energy/100 g vs. energy/portion, (**d**) portion weight vs. energy/portion, (**e**) sugar/100 g vs. sugar/portion, and (**f**) portion weight vs. sugar/portion. Blue dotted lines represent trends for adults’ products.

The scatterplots in Figure 4 show contrary to SRP expectations: (Figure 4a) energy per portion was not associated with sugar/100 g (*r* = 0.056, *p* = 0.127), but (Figure 4b) strongly correlated with sugar/portion (*r* = 0.726, *p* < 0.001); (Figure 4c)) energy/portion was very weakly associated with energy/100 g (*r* = 0.108, *p* = 0.003) but (Figure 4d) was strongly associated with portion weight (*r* = 0.740, *p* < 0.001); (Figure 4e) sugar/portion was more strongly associated with portion weight (*r* = 0.714, *p* < 0.001) than (Figure 4f) with sugar/100 g (*r* = 0.464, *p* < 0.001). Similar, trends were shown for fat (Table 2). After controlling for portion weight, associations with sugar/100 g, energy/100 g, and fat/100 g were strengthened but the association with sugar/portion was weakened (Table 2).

Different trends applied to children’s menus which included child-oriented products such as ice lollies, jellies, and novelty items alongside simpler versions or smaller portions of standard desserts. Apart from sugar/100 g, nutritional parameters had more compact distributions (Figure 4) and lower average values (Supplementary File S4) compared with adults’ products. Unlike adults’ products, energy per portion was moderately correlated with energy/100 g (*r* = 0.671, *p* < 0.001) and with sugar/100 g (*r* = 0.479, *p* < 0.001). Nevertheless, correlations with energy were stronger when nutrients were expressed on a per portion rather than per 100 g basis for both adults’ and children’s desserts (Table 2).

#### 3.3.3. Nutritional Profile in 2020—Comparison of Adults’ and Children’s Products

Children’s desserts contained substantially lower energy and nutrients per portion than adults’ products (Table 3). All comparisons (pooled values for all categories) were highly significant (*p* < 0.001) with moderate effect sizes. Differences were driven by ice-cream and puddings (64% and 20%, respectively, of children’s desserts).

#### 3.3.4. Compliance with Dietary Guidelines

**Figure 5:**
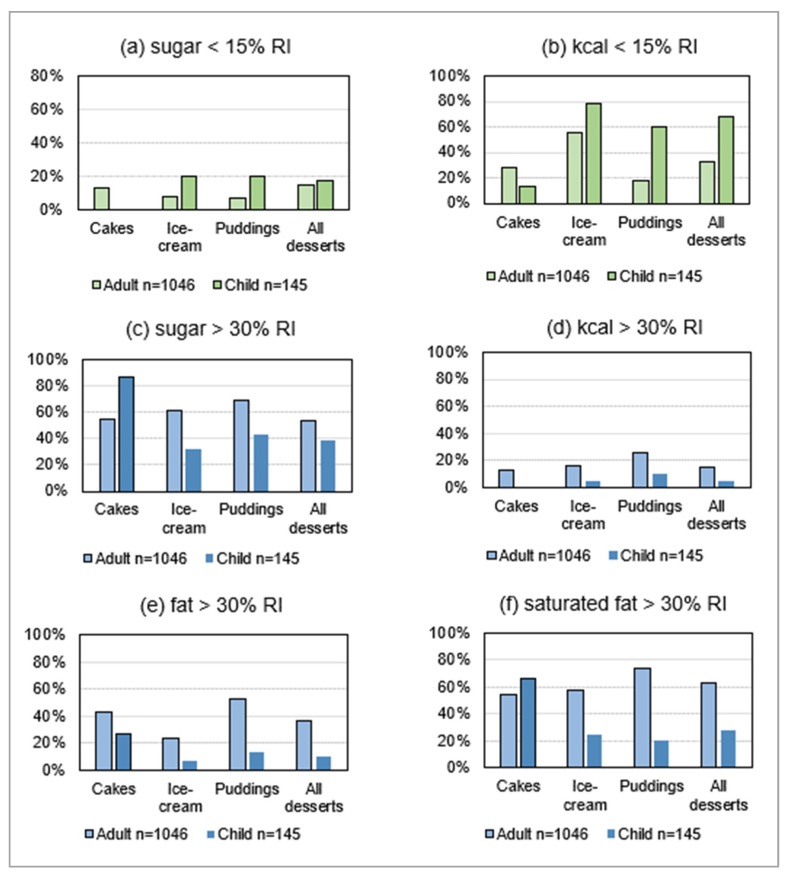
Frequencies of desserts adhering to or exceeding dietary guidelines for selected products on adults’ and children’s menus in 2020. RI: Reference intake—adults’ values refer to EU reference intakes [80]; children’s values are calculated for children aged 2–12 years, from SACN 2011 [82], SACN 2019 [83], and pro-rata from adults’ RI for sugar.

Figure 5 compares frequencies of desserts adhering to/exceeding nutritional guidelines by product and by menu for the principal dessert categories, which represented 79% of adults’ products and 97% of children’s products in 2020.

The energy content was acceptable (<15% RI) in one-third of adults’ desserts and two-thirds of children’s items (Figure 5b), but <20% were low in sugar (Figure 5a). Excessive saturated fat (Figure 5f) was even more pervasive than excessive sugar (Figure 5c) in adults’ products; the puddings category (31% of sample) was the most concerning. Cakes had, by far, the worst nutritional profile among children’s desserts, but represented a minority (10%) of products.

## 4. Discussion

The SRP, a key strand of the governments COP, aimed at reducing sugar and energy in foods. The current investigation is the first independent evaluation of progress towards SRP targets focusing on OOH settings. We have also analysed changes by business as an indication of industry compliance, interrogated the underlying premise that sugar reduction will also lead to energy reduction and compared nutritional values to dietary guidelines to inform priorities for future reformulation programmes. The ultimate objective of reducing the prevalence of excess weight in children is beyond the scope of this study as there is likely to be a lag phase between reformulation and related weight changes. Statistics from the National Child Measurement Programme show an increase in childhood obesity between 2006/2007 and 2019/2020 by age 10–11 years, driven by the most deprived groups [84]. The alarming 4.5 percentage-point increase between 2019 and 2020 is likely to reflect lifestyle changes during the COVID-19 lockdown period.

### 4.1. Summary and Interpretation of Principle Findings

#### 4.1.1. Sugar and Energy Reduction

Our results show statistically significant improvement for ice-cream alone, where 38% sugar reduction (almost double the required amount) was coupled with 25% fat reduction. Together, these changes reduced energy by 30%. In absolute terms, the decreases in sugar (15 g/portion) and energy (97 kcal/portion) compare favourably with the 25 g and 100 kcal/day reductions targeted by the Scientific Advisory Committee on Nutrition. It is notable that the greatest improvements were achieved in a category where increased aeration can be used as an additional tool for reducing sugar and energy per portion, without altering the apparent size of the dessert. Sugar reduction achieved through this route would not be identified by tracking sugar/100 g, and may, partly, explain OHID’s ice-cream results showing 19% decrease in energy/portion but negligible change in sugar/100 g (Table 4).

The magnitude of change for ice-cream contrasts starkly with the modest reductions in cakes, and minimal changes in biscuits and morning goods, implying that technical challenges were harder to overcome in those categories. Indeed, baked goods are among the most difficult products to reformulate without compromising taste, texture, or stability [85,86,87]. The potential for intense sweeteners to assist in sugar reduction is restricted by legislation to products with 30% energy reduction or which contain no added sugar [88]. These requirements are difficult to achieve in products where the bulk and other technical functions of sugar must also be replaced [89]. In practice, few OOH desserts (primarily, ice lollies and jellies) contained sweeteners. These products were only featured on children’s menus, and partly account for the differences between adults’ and children’s products. The (non-significant) increase in puddings may arise from underestimation of baseline values as we could not identify and add nutrition for the customizable toppings.

Table 4 compares our findings with OHID’s final report [49]. Both studies show greatest energy reduction for ice-cream and cakes. OHID found no decrease in sugar/100 g for ice-cream or pooled desserts, despite the decrease in energy. However, the decrease in sugar/portion for pooled desserts found in the present research aligns with findings from Huang and colleagues [50], albeit for a narrower range of products. To our knowledge, no other peer-reviewed study has reported longitudinal data for desserts.

#### 4.1.2. Business Compliance

It is not clear how business performance was calculated in OHID’s final report. Our brand analysis represents changes affecting several food categories, therefore, between-category variability is likely to increase noise and decrease sensitivity for detecting time trends. The four chains which significantly and substantially decreased sugar across their ranges had made changes affecting multiple products and categories. Collectively, they contributed only 12% of ice-creams and 8% of all desserts in 2020. Only one-third of their menu items were ice-creams. The remaining brands included companies with active projects described in PHE’s case studies [73,74] but working with insufficient pace or scale, and others which increased sugar and/or energy, or made no changes at all.

These mixed results demonstrate variable compliance by businesses, as previously observed for retail brands [90]. It is difficult to disentangle lack of commitment from other barriers to reformulation (e.g., limited resources or technical complexity) especially for the ‘semi-active’ brands. The absence of sanctions will presumably have contributed to poor compliance by the inactive brands, but the primary disincentives are likely to include increased costs, consumer rejection of reformulated products, and operational issues, including sourcing and wastage [91,92,93]. As several chains were multinational companies, it is possible that a desire for global brand uniformity might restrict regional reformulation programmes. However, previous research demonstrates significant variability in sugar content for desserts from the same four chains in three countries [94], suggesting some flexibility for local subsidiaries. Providing tailored support and demonstrating consumer acceptance of reformulated or downsized products may be crucial for securing buy-in from businesses.

#### 4.1.3. Validation of Primary Outcome: Correlation between Sugar and Energy 

This investigation, the first to examine correlations between sugar and energy in OOH foods, validates the selection of sugar/portion for monitoring policy compliance. In addition to providing a direct measure of sugar content, our results demonstrate a positive association between sugar/portion and energy/portion, mediated by portion weight. By contrast, sugar/100 g, was not associated with energy/portion in the majority of products, and, therefore, has little relevance as the primary reformulation target in an obesity prevention initiative. These observations are consistent with research on packaged biscuits and cakes that found no correlation between sugar/100 g and energy/100 g [95].

#### 4.1.4. Nutritional Profile in 2020 by Menu

The limited published data on children’s menu items relates to composite meals [96,97]; our research provides the first analysis of children’s desserts. Nutritional values for children’s products were lower than for adults’ both on a per portion basis and in relation to dietary guidelines. However, they were only available in 60% of the brands surveyed. It is notable that sugar/100 g was not lower in children’s products, despite the presence of ‘no added sugar’ claims on many ice lollies, as fruit juice used in place of refined sugar remains a source of free sugars [98]. Nevertheless, through smaller portion sizes, children’s desserts contained 35% less sugar per portion than adults’ products. The ubiquity of low-fat products, where sugar was the main contributor to total energy, might explain the positive correlation between sugar/100 g and energy/portion seen only for children’s products.

At the end of 2020, sugar content was excessive in 54% of adults’ and 39% of children’s desserts. Similarly, saturated fat exceeded the guideline amount in 63% of adults’ and 28% of children’s products which is even more alarming, since desserts are generally not the primary contributor to saturated fat within a meal [76]. These comparisons with dietary guidelines used reference values averaged for ages 2–12 years. Clearly, per portion quantities would have more impact for the younger ages. Likewise, children ordering from the main menu would be exposed to products with even higher quantities in relation to guidelines for their age.

### 4.2. Strengths and Limitations

The current research is the most detailed assessment of desserts from UK chain restaurants, the first to report nutritional values for different types of dessert on both adults’ and children’s menus, and the first specifically designed as an evaluation of SRP. The selected channels represent the most frequently visited eating out locations [99,100] and original data collection in 2020 included more restaurant chains (*n* = 56 vs. *n* = 29) than prior UK research [50] providing a more representative evaluation of the sector. Restaurant screening was based on the most recent market data, eligibility was confirmed from company websites, original end-point data collection followed similar procedures to prior research [76,97,101,102], and robust data-cleaning procedures were implemented prior to analysis. Moreover, our sample included biscuits, cakes, and morning goods (not previously analysed with desserts).

However, limitations must also be acknowledged. Most importantly, a control group was not possible due to the requirement for all food businesses to participate. Consequently, before/after comparisons were the only way to assess programme impact and may have been confounded by prevailing market dynamics. A steady decline in sugar and energy in the five years preceding the OOH baseline (2012–2017) has been demonstrated for household food purchases [103]. Pertinently, 15% reduction in sugar from desserts accounted for 46% of the total decrease [104]. Similar calculations cannot be performed for OOH foods, as conversion factors have not been reviewed since 2013 [105].

Secondly, the impact on population intakes cannot be estimated as there is no source for corresponding sales volumes (the reason for changing OOH outcomes from sales-weighted to simple averages). 

Thirdly, 2017–2020 changes may underestimate reformulation in response to SRP as the revised OOH baseline (August 2017) coincided with the original interim deadline for achieving 5% sugar reduction. Similarly, as baseline data were anonymized, our assessment of brand compliance only captures changes between 2018 and 2020, compounding the lack of sensitivity arising from product diversity and smaller sample size.

Fourthly, reformulation in the final year of the programme was disrupted due to the COVID-19 lockdown from March–June 2020 and subsequent restrictions to daily activities. The condensed core menus available during the data collection period focused on the most popular items—often the most indulgent [106]. They may not have been representative of the full menus. Additionally, bias may have arisen through seasonal variation as the end-point data were collected from September to December, whereas the baseline and interim data were collected throughout the year.

Finally, as in official follow-up and peer-reviewed studies, the published nutritional values were not verified through laboratory analysis and the analysis reflects product availability not what was ordered or consumed by diners.

Generalization to restaurant chains which do not provide nutrition data, smaller chains, or restaurant chains in other countries cannot be assumed, as they may have different characteristics. Dessert chains were a notable omission from the sample; however, they did not publish nutrition data.

### 4.3. Implications for Policy

Successful voluntary policies require: broad coverage; evidence-informed, measurable targets that are realistic within the timescale provided for change; independent monitoring; and transparent reporting [107,108]. These prerequisites were not fulfilled for the OOH sector. The ‘one size fits all’ approach to programme implementation was intended to create a level playing field, but may have underestimated the challenges of data collection from a market sector with few labelling requirements, enabling industry to escape accountability.

Independent monitoring is currently restricted to businesses providing nutritional information on a voluntary basis. In 2020, only half of the potentially eligible restaurant chains screened for inclusion provided nutritional information for desserts. Relatively few (16% of chains screened) provided information on portion weight and/or sugar/100 g, further restricting surveillance. Mandatory calorie labelling [109] was subsequently introduced for large businesses making this a fitting choice as primary outcome for obesity prevention, with expanded coverage. Unless mandatory labelling is extended, monitoring of other nutritional targets is likely to remain incomplete.

A related issue is the basis for reporting nutrient content. Nutrient profiling models in the UK and internationally generally rely on per 100 g measures [110,111,112]. Whilst this is appropriate for pre-packed foods, it was not suitable for the OOH desserts in our survey. Our findings demonstrate that portion weight is the principal determinant of total nutrient and energy content, overwhelming the influence of product composition. Therefore, nutrient labelling, benchmarking criteria, and reformulation targets for the OOH sector should all use per portion metrics, as already adopted for calorie labelling on menus [113] and in the Calorie Reduction Programme [114].

Target setting and guidance also require further consideration. The philosophy of reformulation by stealth underpinning SRP relies on incremental changes which allow consumers time to adapt; it, therefore, restricts the pace of change. However, the category-specific energy targets represented an average decrease of 33% for OOH products [40] which might be difficult to achieve covertly within the three-year timeframe. By comparison, salt reduction has taken 10 years [115]. Some categories may require a longer implementation period for changes of this magnitude. As each component of a dessert will contribute to the overall nutritional profile, suppliers to the hospitality industry have an important role and may be crucial for helping smaller chains with less development capability.

Reducing sugar and saturated fat should both be considered high priorities in future initiatives. Moreover, portion size should have greater emphasis as an independent target for intervention with potential for recalibrating social norms and reducing exposure to all nutrients of concern. Consistent evidence shows that reducing the availability of large portions has the potential to reduce energy intake by 12–16% [116].

Menu engineering could be used to achieve these nutritional goals in hospitality settings. Restaurants are already delisting some of the most extreme menu items and offering mini desserts alongside the standard portion size, implying acceptability to both businesses and consumers (unlike ‘shrinkflation’—where downsizing is interpreted as reducing value for money). This could be extended by increasing the range of smaller and/or less indulgent options (e.g., fruit-based or aerated desserts) while reformulating other products more gradually. In addition, as high availability alone is insufficient to override hedonic cues, salience and desirability should be emphasized through menu prominence and descriptions/photographs that emphasize deliciousness rather than health [117].

## 5. Conclusions

The present research provides valuable information to support the development of policies in a rapidly growing arena with acknowledged data and implementation gaps.

Our findings demonstrate that policy targets were achieved in one of five categories and by a minority of restaurant chains. These results imply that voluntary measures can drive change when reformulation is achieved at reasonable cost without compromising consumer acceptance. However, business engagement was variable. Further research should investigate barriers to progress in other categories and assist in developing solutions, giving highest priority to puddings which contain excessive amounts of sugar, fat, and saturated fat. Stronger levers may be required to ensure sustained industry commitment.

Our findings also highlight the importance of tailoring interventions for each setting; specifically selecting measurable outcomes and effective implementation methods. Energy/portion is the only measurable nutrient-based outcome for OOH foods unless mandatory labelling requirements are extended. The pivotal role of portion weight identified in this study suggests that interventions addressing portion size will enable faster sugar and energy reduction than achievable through the gradual recipe adjustment required for silent reformulation. Menu architecture must also promote consumer demand.

## Figures and Tables

**Table 1:** Number of products by year, stratified by menu for 2018 and 2020.

Product Category	Menu	2017 ^1^		2018		2020		Total	
		N	%	N	%	N	%	N	%
Biscuits	Adult’s			70		101		172	
	Child’s			4		5		9	
	Total	49	5.9%	74	5.1%	106	8.9%	229	6.6%
Cake	Adult’s			328		318		646	
	Child’s			18		15		33	
	Total	216	26.2%	346	23.8%	333	28.0%	895	25.8%
Ice-cream	Adult’s			262		172		434	
	Child’s			147		95		242	
	Total	250	30.3%	409	28.2%	267	22.4%	926	26.7%
Morning goods	Adult’s			104		114		218	
	Child’s			0		0		0	
	Total	93	11.3%	104	7.2%	114	9.6%	311	9.0%
Puddings	Adult’s			466		341		807	
	Child’s			52		30		82	
	Total	216	26.2%	518	35.7%	371	31.2%	1105	31.9%
Total	Adult’s			1230		1046		2277	
	Child’s			221		145		366	
	Total	824	100%	1451	100%	1191	100%	3466	100%

^1^ Children’s desserts not identified in 2017 dataset.

**Table 2:** Correlations of nutrients and portion weight with energy per portion in adults’ and children’s desserts.

Menu	Control Variable	Correlation with kcal/portion	Sugar g/100 g	Fat g/100 g	Energy kcal/100 g	Sugar g/portion	Fat g/portion	Portion Weight (g)	Kcal per Portion
Adult’s	None	Pearson’s *r*	0.056	0.205	0.108	0.726	0.872	0.74	1.000
		Significance ^2^	0.127	**<0.001**	**0.003**	**<0.001**	**<0.001**	**<0.001**	
		*df*	749	749	749	749	749	749	0
	Portion weight	Pearson’s *r*	0.29	0.712	0.834	0.42	0.857		1.000
		Significance ^2^	**<0.001**	**<0.001**	**<0.001**	**<0.001**	**<0.001**		
		*df*	748	748	748	748	748		0
Child’s	None	Pearson’s *r*	0.479	0.621	0.671	0.825	0.869	0.456	1.000
		Significance ^2^	**<0.001**	**<0.001**	**<0.001**	**<0.001**	**<0.001**	**0.001**	
		*df*	48	48	48	48	48	48	0.000
	Portion weight	Pearson’s *r*	0.717	0.808	0.913	0.775	0.87		1.000
		Significance ^2^	**<0.001**	**<0.001**	**<0.001**	**<0.001**	**<0.001**		
		*df*	47	47	47	47	47		0

^2^ Significant *p*-values in bold text.

**Table 3:** Comparison of nutritional values (g or kcal per portion) in adults’ and children’s desserts 2020.

Category	Nutrient	Menu	N	Median	IQR	% Diff	*p*-Value ^3^	Effect Size *r*
All desserts	Sugar	Adult’s	1046	28.7	18.1–42.1			
		Child’s	145	18.6	12.6–28.5	−35%	**<0.001**	−0.18
	kcal	Adult’s	1046	372	265–513			
		Child’s	145	171	94–272	−54%	**<0.001**	−0.37
	Fat	Adult’s	1046	17.6	11.0–25.0			
		Child’s	145	5.9	2.7–9.9	−67%	**<0.001**	−0.37
	Saturated fat	Adult’s	1046	7.8	4.2–12.5			
		Child’s	145	3.1	1.0–5.5	−60%	**<0.001**	−0.31
Biscuits	Sugar	Adult’s	101	19.4	13.0–29.0			
		Child’s	5	14.2	9.2–19.2	−27%	0.177	−0.13
	kcal	Adult’s	101	273	199–356			
		Child’s	5	181	109–239	−34%	**0.030**	−0.21
	Fat	Adult’s	101	13.2	8.5–18.0			
		Child’s	5	4.8	2.4–6.2	−64%	**0.001**	−0.31
	Saturated fat	Adult’s	101	6.6	3.9–9.3			
		Child’s	5	1.8	1.0–2.7	−73%	**0.002**	−0.30
Cake	Sugar	Adult’s	318	28.7	21.5–39 7			
		Child’s	15	32.5	27.2–47.2	13%	0.180	0.07
	kcal	Adult’s	318	388	292–488			
		Child’s	15	353	291–392	−9%	0.082	−0.12
	Fat	Adult’s	318	19.6	12.9–25.1			
		Child’s	15	14.3	13.8–18.9	−27%	**0.048**	−0.11
	Saturated fat	Adult’s	318	6.6	3.7–11.7			
		Child’s	15	5.5	4.4–6.5	−17%	0.392	−0.05
Ice-cream	Sugar	Adult’s	172	32.9	20.1–51.8			
		Child’s	95	16.6	11.9–24.0	−49%	**<0.001**	−0.43
	kcal	Adult’s	172	275	164–475			
		Child’s	95	117	94–214	−58%	**<0.001**	−0.46
	Fat	Adult’s	172	11.8	6.0–19.9			
		Child’s	95	4.6	1.3–8.5	−61%	**<0.001**	−0.41
	Saturated fat	Adult’s	172	7.0	3.9–11.9			
		Child’s	95	3.1	0.8–4.9	−55%	**<0.001**	−0.41
Morning goods	Sugar	Adult’s	114	14.5	8.5–20.1	-	-	-
		Child’s	0	-	-			
	kcal	Adult’s	114	324	270–381	-	-	-
		Child’s	0	-	-			
	Fat	Adult’s	114	14.4	10.8–18.2	-	-	-
		Child’s	0	-	-			
	Saturated fat	Adult’s	114	8.5	4.3–10.3			
		Child’s	0	-	-			
Puddings	Sugar	Adult’s	341	35.8	24.0–54.7			
		Child’s	30	18.8	15.3–28.8	−48%	**<0.001**	−0.24
	kcal	Adult’s	341	471	350–606			
		Child’s	30	194	72–274	−59%	**<0.001**	−0.34
	Fat	Adult’s	341	22.1	14.8–29.2			
		Child’s	30	5.7	0.9–7.7	−74%	**<0.001**	−0.36
	Saturated fat	Adult’s	341	10.5	5.9–16.0			
		Child’s	30	2.6	0.4–4.3	−75%	**<0.001**	−0.32

^3^ Mann–Whitney test comparing nutrition for adults’ and children’s desserts (significant *p*-values in bold text).

**Table 4:** Percentage changes in sugar and energy compared with other studies.

	This Study 2017 vs. 2020 (Two Years’ Data)		OHID [49] 2017 vs. 2020 (Two Years’ Data)		Huang et al. [50] 2018–2020 (Three Years’ Data)	
	Sugar /portion (*n* = 2642)	Kcal /portion (*n* = 2642)	Sugar /100 g (*n* = 1478)	Kcal /portion (*n* = 2930)	Sugar /portion (*n* = 1536)	Kcal /portion (*n* = 1536)
Biscuits	0%	−3%	0%	3%		
Cakes	−9%	−10%	−8%	−16%		
Ice-cream	−38%	−30%	1%	−19%		
Morning goods	4%	1%	−4%	−2%		
Puddings	6%	6%	0%	−9%		
All desserts	−11%	−4%	0%	−11%	−16%	−19%

## Data Availability

Data can be obtained from the corresponding author.

## Supplementary Materials

The following supporting information can be downloaded at: https://www.mdpi.com/article/10.3390/nu15143149/s1, Supplementary File S1: Table S1. Product categorisation; Supplementary File S2: Table S2. Restaurants included in the study; Supplementary File S3: Figure S1. Distributions of sugar, energy, fat, and saturated fat by category and year; Supplementary File S4: Table S3. Descriptive statistics for subset with per 100 g data.

## References

[1] NHS Digital (2020). Health Survey for England 2019.

[2] World Health Organization Obesity and Overweight Fact-Sheet.

[3] Abarca-Gómez L., Abdeen Z.A., Hamid Z.A., Abu-Rmeileh N.M., Acosta-Cazares B., Acuin C., Adams R.J., Aekplakorn W., Afsana K., Aguilar-Salinas C.A. (2017). Worldwide trends in body-mass index, underweight, overweight, and obesity from 1975 to 2016: A pooled analysis of 2416 population-based measurement studies in 128.9 million children, adolescents, and adults. Lancet.

[4] Rankin J., Matthews L., Cobley S., Han A., Sanders R., Wiltshire H.D., Baker J.S. (2016). Psychological consequences of childhood obesity: Psychiatric comorbidity and prevention. Adolesc. Health Med. Ther..

[5] Morales Camacho W.J., Molina Díaz J.M., Plata Ortiz S., Plata Ortiz J.E., Morales Camacho M.A., Calderón B.P. (2019). Childhood obesity: Aetiology, comorbidities, and treatment. Diabetes Metab. Res..

[6] Candler T.P., Mahmoud O., Lynn R.M., Majbar A.A., Barrett T.G., Shield J. (2018). Continuing rise of type 2 diabetes incidence in children and young people in the UK. Diabet. Med..

[7] Caprio S., Santoro N., Weiss R. (2020). Childhood obesity and the associated rise in cardiometabolic complications. Nat. Metab..

[8] Geserick M., Vogel M., Gausche R., Lipek T., Spielau U., Keller E., Pfäffle R., Kiess W., Körner A. (2018). Acceleration of BMI in early childhood and risk of sustained obesity. N. Engl. J. Med..

[9] Stock K., Nagrani R., Gande N., Bernar B., Staudt A., Willeit P., Geiger R., Knoflach M., Kiechl-Kohlendorfer U., Winder B. (2020). Birth Weight and Weight Changes from Infancy to Early Childhood as Predictors of Body Mass Index in Adolescence. J. Pediatr..

[10] Ryder J.R., Jacobs D.R., Sinaiko A.R., Kornblum A.P., Steinberger J. (2019). Longitudinal Changes in Weight Status from Childhood and Adolescence to Adulthood. J. Pediatr..

[11] Reilly J.J., Kelly J. (2011). Long-term impact of overweight and obesity in childhood and adolescence on morbidity and premature mortality in adulthood: Systematic review. Int. J. Obes..

[12] World Health Organization Report of the Commission on Ending Childhood Obesity. https://www.who.int/end-childhood-obesity/final-report/en/.

[13] World Obesity Federation Our Policy Priorities.

[14] Davies S.C. (2019). Time to Solve Childhood Obesity: An Independent Report by the Chief Medical Officer, 2019.

[15] Jebb S.A., Aveyard P.N., Hawkes C. (2013). The evolution of policy and actions to tackle obesity in England. Obes. Rev..

[16] Adams J., Mytton O., White M., Monsivais P. (2016). Why are some population interventions for diet and obesity more equitable and effective than others? The role of individual agency. PLoS Med..

[17] Dahlgren G., Whitehed M. (2006). European Strategies for Tackling Social Inequities in Health.

[18] Nobles J., Summerbell C., Brown T., Jago R., Moore T. (2021). A secondary analysis of the childhood obesity prevention Cochrane Review through a wider determinants of health lens: Implications for research funders, researchers, policymakers and practitioners. Int. J. Behav. Nutr. Phys. Act..

[19] Theis D., White M. (2021). Is Obesity Policy in England Fit for Purpose? Analysis of Government Strategies and Policies, 1992–2020. Milbank Q..

[20] Rutter H., Bes-Rastrollo M., De Henauw S., Lahti-Koski M., Lehtinen-Jacks S., Mullerova D., Rasmussen F., Rissanen A., Visscher T.L., Lissner L. (2017). Balancing upstream and downstream measures to tackle the obesity epidemic: A position statement from the european association for the study of obesity. Obes. Facts.

[21] Butland B., Jebb S., Kopelman P., McPherson K., Thomas S., Mardell J., Parry V. (2007). Foresight. Tackling obesities: Future choices. Project report. Obes. Rev..

[22] Lobstein T., Brinsden T., Neveux M. World Obesity Atlas 2022. https://www.worldobesityday.org/assets/downloads/World_Obesity_Atlas_2022_WEB.pdf.

[23] Di Cesare M., Sorić M., Bovet P., Miranda J.J., Bhutta Z., Stevens G.A., Laxmaiah A., Kengne A., Bentham J. (2019). The epidemiological burden of obesity in childhood: A worldwide epidemic requiring urgent action. BMC Med..

[24] Ritchie H., Roser M. (2017). Obesity. https://ourworldindata.org/obesity.

[25] Harris J.L., Pomeranz J.L., Lobstein T., Brownell K.D. (2009). A crisis in the marketplace: How food marketing contributes to childhood obesity and what can be done. Annu. Rev. Public Health.

[26] Russell S.J., Croker H., Viner R.M. (2019). The effect of screen advertising on children’s dietary intake: A systematic review and meta-analysis. Obes. Rev..

[27] Benson C. (2009). Increasing portion size in Britain. Soc. Biol. Hum. Aff..

[28] Are We Suffering from Portions Distortion?. https://www.which.co.uk/news/2020/09/are-we-suffering-from-portion-distortion/.

[29] Burgoine T., Sarkar C., Webster C.J., Monsivais P. (2018). Examining the interaction of fast-food outlet exposure and income on diet and obesity: Evidence from 51,361 UK Biobank participants. Int. J. Behav. Nutr. Phys. Act..

[30] Cetateanu A., Jones A. (2014). Understanding the relationship between food environments, deprivation and childhood overweight and obesity: Evidence from a cross sectional England-wide study. Health Place.

[31] United Nations Children’s Fund (UNICEF) (2019). *Prevention of Overweight and Obesity in Children and Adolescents:* UNICEF Programming Guidance. https://www.unicef.org/media/92336/file/Programming-Guidance-Overweight-Prevention.pdf.

[32] Gressier M., Sassi F., Frost G. (2020). Healthy foods and healthy diets. How government policies can steer food reformulation. Nutrients.

[33] Te Morenga L., Mallard S., Mann J. (2013). Dietary sugars and body weight: Systematic review and meta-analyses of randomised controlled trials and cohort studies. BMJ.

[34] Turck D., Bohn T., Castenmiller J., de Henauw S., Hirsch-Ernst K.I., Knutsen H.K., Maciuk A., Mangelsdorf I., McArdle H.J., EFSA Panel on Nutrition, Novel Foods and Food Allergens (NDA) (2022). Tolerable upper intake level for dietary sugars. EFSA J..

[35] Malik V.S., Hu F.B. (2022). The role of sugar-sweetened beverages in the global epidemics of obesity and chronic diseases. Nat. Rev. Endocrinol..

[36] World Health Organization (2015). Guideline: Sugars Intake for Adults and Children.

[37] Scientific Advisory Committee on Nutrition (SACN) (2015). Carbohydrates and Health.

[38] House of Commons Health Committee (2015). Childhood Obesity—Brave and Bold Action.

[39] HM Government (2016). Childhood Obesity: A Plan for Action.

[40] Tedstone A., Targett V., Allen R. (2015). Sugar Reduction: The Evidence for Action.

[41] Tedstone A. (2018). The World’s First Sugar Reduction Programme: Data Challenges.

[42] WHO Regional Office for Europe (2020). Improving Dietary Intake and Achieving Food Product Improvement Policy Opportunities and Challenges for the WHO European Region in Reducing Salt and Sugar in the Diet.

[43] Tedstone A., Targett V., Owtram G., Pyne V., Allen R., Bathrellou K., MacKinlay B., Kathryn E., Morgan K., Swan G. (2017). Sugar Reduction: Achieving the 20%.

[44] Caraher M., Perry I. (2017). Sugar, salt, and the limits of self regulation in the food industry. BMJ.

[45] Knai C., Petticrew M., Mays N. (2016). The childhood obesity strategy. BMJ.

[46] Buttriss J.L. (2016). Sugars—Part of a bigger picture?. Nutr. Bull..

[47] Griffith R., Jenneson V., James J., Taylor A. (2021). The Impact of a Tax on Added Sugar and Salt.

[48] Department of Health and Social Care (2021). Press Release. UK to Spearhead Europe-Wide Initiative to Reduce Sugar and Calorie Intake in Food.

[49] Office for Health Improvement & Disparities (2022). Sugar Reduction Programme: Industry Progress 2015 to 2020.

[50] Huang Y., Theis D.R., Burgoine T., Adams J. (2021). Trends in energy and nutrient content of menu items served by large UK chain restaurants from 2018 to 2020: An observational study. BMJ Open.

[51] (2018). UK Statutory Instruments No. 41. The Soft Drinks Industry Levy Regulations 2018. https://www.legislation.gov.uk/uksi/2018/41/contents/made.

[52] Bakery Cafes in the UK. https://www.ibisworld.com/united-kingdom/market-research-reports/bakery-cafes-industry/.

[53] Takeaway & Fast-Food Restaurants in the UK. https://www.ibisworld.com/united-kingdom/market-research-reports/takeaway-fast-food-restaurants-industry/.

[54] Cafes & Coffee Shops in the UK. https://www.ibisworld.com/united-kingdom/market-research-reports/cafes-coffee-shops-industry/.

[55] Full-Service Restaurants in the UK. https://www.ibisworld.com/united-kingdom/market-research-reports/full-service-restaurants-industry/.

[56] Juice & Smoothie Bars in the UK—Market Research Report. https://www.ibisworld.com/united-kingdom/market-research-reports/juice-smoothie-bars-industry/.

[57] Catering Services in the UK—Market Research Report. https://www.ibisworld.com/united-kingdom/market-research-reports/catering-services-industry/.

[58] Food Markets in the UK—Market Research Report. https://www.ibisworld.com/united-kingdom/market-research-reports/food-markets-idustry/.

[59] Online Food Ordering & Delivery Platforms in the UK. https://www.ibisworld.com/united-kingdom/market-research-reports/online-food-ordering-delivery-platforms-industry/.

[60] Bakery Product Retailing in the UK. https://www.ibisworld.com/united-kingdom/market-research-reports/bakery-product-retailing-industry/.

[61] Government of Ontario Healthy Menu Choices Act, 2015. S.O. 2015, c. 7, Sched. 1 2016. https://www.ontario.ca/laws/statute/15h07.

[62] Food and Drug Administration, HHS (2014). Food labeling; nutrition labeling of standard menu items in restaurants and similar retail food establishments. Final rule. Fed. Regist..

[63] Top 100 UK Operator Profiles. https://www.mca-insight.com/market-intelligence/operator-data-index/top-100-profiles.

[64] The Most Popular Dining Brands in the UK. https://yougov.co.uk/ratings/food/popularity/dining-brands/all.

[65] Most Popular Dining Brands in the UK as of June 2019. https://www.statista.com/statistics/950444/most-popular-restaurant-brands-in-the-united-kingdom-uk/.

[66] Leading 25 Retailers of Baked Products in the United Kingdom (UK) in 2016, Ranked by Number of Outlets. https://www.statista.com/statistics/297842/leading-retailers-of-baked-products-in-the-united-kingdom-uk/.

[67] Leading Coffee Shop Chains Ranked by Number of Outlets in the United Kingdom (UK) as of December 2016. https://www.statista.com/statistics/297863/leading-coffeehttps://www.statista.com/statistics/297863/leading-coffee-shop-chains-in-the-united-kingdom-uk-store-number/#:~:text=Costa%20ranked%20highest%20with%202%2C121,in%20the%20last%208%20years-s.

[68] Leading Casual Dining Brands in the United Kingdom (UK) as of December 2016 and December 2017, by Number of Units. https://www.statista.com/statistics/629796/casual-dining-brands-by-number-of-units-united-kingdom-uk/.

[69] Leading Restaurant Chains Ranked by Number of Users in Great Britain from 2018. https://www.statista.com/statistics/586234/restaurant-chains-usage-in-the-uk-by-number-of-users/.

[70] Tedstone A. (2017). Sugar Reduction Timeline.

[71] Coyle N. (2020). Dataset OOH_2017.

[72] Coyle N. (2020). Dataset OOH_2018.

[73] Tedstone A., Coulton V., Targett V., Bennett A., Sweeney K., Morgan K., Morgan K., Clegg E., Robinson M., Dowd L. (2018). Sugar Reduction and Wider Reformulation Programme: Progress towards the First 5% Reduction and Next Steps.

[74] Niblett P., Coyle N., Little E., Beaton C., Burton J., Chisholm S., Tedstone A., Targett V., Nicholas J., Montel S. (2019). Sugar Reduction: Report on Progress between 2015 and 2018.

[75] Coyle N., Little E., Williamson S., Dodhia S., Targett V., Montel S., Niblett P., Mildon A., Hutchinson K., Owtram G. (2020). Sugar Reduction: Report on Progress between 2015 and 2019.

[76] Theis D.R., Adams J. (2019). Differences in energy and nutritional content of menu items served by popular UK chain restaurants with versus without voluntary menu labelling: A cross-sectional study. PLoS ONE.

[77] IBM Corp (2021). IBM SPSS Statistics for Windows.

[78] Ghasemi A., Zahediasl S. (2012). Normality tests for statistical analysis: A guide for non-statisticians. Int. J. Endocrinol. Metab..

[79] Fritz C.O., Morris P.E., Richler J.J. (2012). Effect Size Estimates: Current Use, Calculations, and Interpretation. J. Exp. Psychol. Gen..

[80] (2011). Regulation (EU) No 1169/2011 of the European Parliament and of the Council of 25 October 2011 on the provision of food information to consumers. Off. J. Eur. Union..

[81] Public Health England (2017). Encouraging Healthier ‘Out of Home’ Food Provision.

[82] Scientific Advisory Committee on Nutrition (SACN) Dietary Reference Values for Energy.

[83] Scientific Advisory Committee on Nutrition (2019). Saturated Fats and Health.

[84] NHS Digital National Child Measurement Programme, England 2020/21 School Year.

[85] Biguzzi C., Schlich P., Lange C. (2014). The impact of sugar and fat reduction on perception and liking of biscuits. Food Qual. Prefer..

[86] Milner L., Kerry J.P., O’Sullivan M.G., Gallagher E. (2020). Physical, textural and sensory characteristics of reduced sucrose cakes, incorporated with clean-label sugar-replacing alternative ingredients. Innov. Food Sci. Emerg. Technol..

[87] Van der Sman R., Renzetti S. (2020). Understanding functionality of sucrose in cake for reformulation purposes. Crit. Rev. Food Sci. Nutr..

[88] (2004). UK Statutory Instruments No. 3348. *The Sweeteners in Food (Amendment) (England) Regulations 2004*. https://www.legislation.gov.uk/uksi/2004/3348/made.

[89] Cooper J. (2017). Food and drink reformulation to reduce fat, sugar and salt. Food Sci. Technol..

[90] Bandy L.K., Scarborough P., Harrington R.A., Rayner M., Jebb S.A. (2021). The sugar content of foods in the UK by category and company: A repeated cross-sectional study, 2015–2018. PLoS Med..

[91] Glanz K., Resnicow K., Seymour J., Hoy K., Stewart H., Lyons M., Goldberg J. (2007). How Major Restaurant Chains Plan Their Menus: The Role of Profit, Demand, and Health. Am. J. Prev. Med..

[92] World Health Organization (2017). Incentives and Disincentives for Reducing Sugar in Manufactured Foods: An Exploratory Supply Chain Analysis: A Set of Insights for Member States in the Context of the WHO European Food and Nutrition Action Plan 2015–2020.

[93] Fuster M., Handley M.A., Alam T., Fullington L.A., Elbel B., Ray K., Huang T.T.-K. (2021). Facilitating Healthier Eating at Restaurants: A Multidisciplinary Scoping Review Comparing Strategies, Barriers, Motivators, and Outcomes by Restaurant Type and Initiator. Int. J. Environ. Res. Public Health.

[94] Hobin E., White C., Li Y., Chiu M., O’Brien M.F., Hammond D. (2014). Nutritional quality of food items on fast-food ‘kids’ menus’: Comparisons across countries and companies. Public Health Nutr..

[95] Alessandrini R., He F.J., Hashem K.M., Tan M., MacGregor G.A. (2019). Reformulation and priorities for reducing energy density; Results from a cross-sectional survey on fat content in pre-packed cakes and biscuits sold in British supermarkets. Nutrients.

[96] Reeves S., Wake Y., Zick A. (2011). Nutrition labeling and portion size information on children’s menus in fast-food and table-service chain restaurants in London, UK. J. Nutr. Educ. Behav..

[97] Young M., Coppinger T., Reeves S. (2019). The Nutritional Value of Children’s Menus in Chain Restaurants in the United Kingdom and Ireland. J. Nutr. Educ. Behav..

[98] Swan G.E., Powell N.A., Knowles B.L., Bush M.T., Levy L.B. (2018). A definition of free sugars for the UK. Public Health Nutr..

[99] The Food and You Survey Wave 5. https://www.food.gov.uk/research/food-and-you/food-and-you-wave-five.

[100] Avison Z. (2019). Foodservice Insights: Eating-Out Review 2019.

[101] Robinson E., Jones A., Whitelock V., Mead B.R., Haynes A. (2018). (Over)eating out at major UK restaurant chains: Observational study of energy content of main meals. BMJ.

[102] Muc M., Jones A., Roberts C., Sheen F., Haynes A., Robinson E. (2019). A bit or a lot on the side? Observational study of the energy content of starters, sides and desserts in major UK restaurant chains. BMJ Open.

[103] Berger N., Cummins S., Smith R.D., Cornelsen L. (2019). Recent trends in energy and nutrient content of take-home food and beverage purchases in Great Britain: An analysis of 225 million food and beverage purchases over 6 years. BMJ Nutr. Prev. Health.

[104] Cornelsen L., Berger N., Smith R., Cummins S. (2018). OP41 Nutritional content of household food purchases: Study of trends and socio-economic inequalities in Britain 2012–2017. J. Epidemiol. Community Health.

[105] Defra Statistics (2020). Family Food 2018/19. https://www.gov.uk/government/statistics/family-food-201819.

[106] Hancock A., Evans J. (2020). Consumers Sweet on Desserts as Pandemic Spurs Home Deliveries. Financial Times. https://www.ft.com/content/a7ae1c3c-bb68-499b-9664-441902a10fe5.

[107] Knai C., Petticrew M., Douglas N., Durand M.A., Eastmure E., Nolte E., Mays N. (2018). The public health responsibility deal: Using a systems-level analysis to understand the lack of impact on alcohol, food, physical activity, and workplace health sub-systems. Int. J. Environ. Res. Public Health.

[108] Mozaffarian D., Angell S.Y., Lang T., Rivera J.A. (2018). Role of government policy in nutrition—Barriers to and opportunities for healthier eating. BMJ.

[109] Department of Health & Social Care Mandating Calorie Labelling in the Out-of-Home Sector Government Response to Public Consultation.

[110] Department of Health (2011). Nutrient Profiling Technical Guidance.

[111] Poon T., Labonté M., Mulligan C., Ahmed M., Dickinson K.M., L’Abbé M.R. (2018). Comparison of nutrient profiling models for assessing the nutritional quality of foods: A validation study. Br. J. Nutr..

[112] WHO Regional Office for Europe Nutrient Profile Model: Second Edition.

[113] Department of Health & Social Care Calorie Labelling in the Out of Home Sector: Implementation Guidance.

[114] Pyne V., Montel S., Targett V., Little E., Owtram G., Tedstone A., O’Kennedy E. Calorie Reduction Technical Report: Guidelines for Industry, 2017 Baseline in Key Foods and Next Steps.

[115] Public Health England (2018). Salt Targets 2017: Progress Report. A Report on the Food Industry’s Progress towards Meeting the 2017 Salt Targets.

[116] Hollands G.J., Shemilt I., Marteau T.M., Jebb S.A., Lewis H.B., Wei Y., Higgins J.P., Ogilvie D. (2015). Portion, package or tableware size for changing selection and consumption of food, alcohol and tobacco. Cochrane Database Syst. Rev..

[117] Bauer J.M., Nielsen K.S., Hofmann W., Reisch L.A. (2022). Healthy eating in the wild: An experience-sampling study of how food environments and situational factors shape out-of-home dietary success. Soc. Sci. Med..

[118] Moher D., Liberati A., Tetzlaff J., Altman D.G., Prisma Group (2009). Preferred reporting items for systematic reviews and meta-analyses: The PRISMA statement. PLoS Med..

