# Supplementary material for: Tackling (Childhood) Obesity through a Voluntary Food Reformulation Policy: A Repeated Cross-Sectional Study Investigating Nutritional Changes in the Out-of-Home Sector
Source: Nutrients. 2023 Jul 14;15(14):3149. doi: 10.3390/nu15143149 (PMC10384819; doi:10.3390/nu15143149)
Supplement: Supplementary file 1 [file nutrients-15-03149-s001.zip › Supplementary File S1. Product categorisation.pdf]

Supplementary File S1

Table S1. Product categorisation

Category	Source	Products
Biscuits	PHE	All types of sweet biscuits, cereal bars and toaster pastries; breakfast biscuits; rice cakes; gluten free biscuits; in-store bakery products
	MCA	Biscuit & cookie; cookie; gingerbread; shortbread; fruit bar cereal bar; cereal bar/flapjack; confectionery/biscuit; granola
Cakes	PHE	All types of cakes, ambient and chilled, including cake bars and slices
	MCA	Brownie; Bakewell bar slice; almond bar slice; caramel millionaires billionaires bar slice; churros; rocky road bar slice; cake - total; tiffin; traybake cake/caramel bar/rocky road/flapjack/almond slice etc.; traybake/slice/bar/bite; energy slice bar bite; muffin sweet; muffin; flapjack; cake/muffin/tart
Ice cream	PHE	All types of ice cream, dairy and non-dairy, choc ices, ice cream desserts, milk ice lollies, ice lollies; low fat/low calorie ice cream; sorbet; frozen yogurt
	MCA	Frozen yogurt; ice cream - ice cream; sorbet; ice cream – sundae
Morning Goods	PHE	Includes croissants, crumpets, English muffins, buns, teacakes, scones, waffles, Danish pastries, fruit loaves, bagels
	MCA	Bagel - sweet; Belgian bun; breakfast loaf; bun, bread, pancake, scone & waffle; cinnamon bun roll swirl; croissant; croissant sweet - total including pain au and twist; crepe; crumpet; Danish pastry; Danish pastry/doughnut; fruit bread fruit toast; iced bun; scone; teacake; teacake Welsh cake; waffle; toasted muffin
Puddings	PHE	All types of ambient, chilled and frozen large and individual pies, tarts and flans, cheesecake, gateaux, dairy desserts, sponge and rice puddings.<br>Also in data file: Pancakes, waffles, pastel de nata, fruit salad, custard, freakshake cake, warm brownie with ice cream, warm cookie dough sandwich with ice cream, chocolate fudge cake
	MCA	Cheesecake - total; crème brulee; crème caramel; crumble; eclair choux or profiterole; Eton mess; meringue-based dessert; mousse; panna cotta; pie sweet - total; posset; rice pudding; soufflé; sticky toffee pudding; tart sweet - total; tiramisu; trifle; cold dessert; hot dessert; other dessert; pie/tart; jelly; fondant
