# Supplementary material for: Tackling (Childhood) Obesity through a Voluntary Food Reformulation Policy: A Repeated Cross-Sectional Study Investigating Nutritional Changes in the Out-of-Home Sector
Source: Nutrients. 2023 Jul 14;15(14):3149. doi: 10.3390/nu15143149 (PMC10384819; doi:10.3390/nu15143149)
Supplement: Supplementary file 1 [file nutrients-15-03149-s001.zip › Supplementary File S2. Restaurants included in the study.pdf]

Supplementary File S2

Table S2. Restaurants included in the study

**Brand**	**Channel**	**Outlets 2020**	**Year 2018 or 2020**	**Reason for exclusion in 2020**
All Bar One	FSR	56	Both	
Ask Italian	FSR	66	Both	
Beefeater	FSR	165	Both	
Bella Italia	FSR	78	2020	
Bill's	FSR	80	Both	
Birds Bakery	CCB	62	2020	
Brewers Fayre	FSR	160	Both	
Burger King	FF	530	Both	
Café Rouge	FSR	30	2020	
Caffe Nero	CCB	672	Both	
Chef & Brewer	FSR	140	Both	
Chicken Cottage	FF	47	Both	
Chipotle	QSR	8	2018	< 20 outlets
Chop'd	QSR	13	2018	< 20 outlets
Coco di Mama	FF	35	Both	
Coffee Republic	CCB	30	2020	
Coffee#1	CCB	100	2020	
Coopland & Son	CCB	171	2020	
Costa	CCB	2400	Both	
Crepe Affaire	CCB	17	2018	< 20 outlets
Crown Carveries	FSR	90	2018	No nutrition
Crussh	FC	35	Both	
Dominos	FF	1126	Both	
EAT	QSR	0	2018	Ceased trading
Eating Inn	FSR	0	2018	Ceased trading
Ember Inns	FSR	138	Both	
Farmhouse Inns	FSR	72	2018	No nutrition
Fayre & Square	FSR	0	2018	Ceased trading
Flaming Grill	FSR	>20	Both	
Generous George	FSR	20	Both	
Gourmet Burger Kitchen	FSR	60	Both	
Greggs	CCB	1953	Both	
Gusto	FSR	13	2018	< 20 outlets
Harvester	FSR	230	Both	
Hungry Horse	FSR	270	Both	
Itsu	FC	74	Both	
Jamie's Italian	FSR	0	2018	Ceased trading
John Barras	FSR	0	2018	Ceased trading
Kentucky Fried Chicken	FF	900	Both	

Krispy Kreme	CCB	130	Both	
Leon	FC	65	Both	
Lloyds No. 1	FSR	1	2018	< 20 outlets
Loch Fyne	FSR	10	2018	< 20 outlets
McDonalds	FF	1300	Both	
Nando's	FSR	340	Both	
Old English Inns	FSR	100	2018	No nutrition
O'Neill's	FSR	49	2018	No nutrition
Papa john's	FF	450	Both	
Paul	CCB	37	Both	
Pizza Express	FSR	470	Both	
Pizza Hut	FF	215	Both	
Pod	QSR	6	2018	< 20 outlets
Pret	CCB	367	Both	
Prezzo	FSR	180	2020	
Pure	FC	22	Both	
Shake Shack	QSR	17	2018	< 20 outlets
Sizzling Pubs	FSR	>20	Both	
Slug & Lettuce	FSR	83	2018	No nutrition
Soho Coffee Co	CCB	35	Both	
Starbucks	CCB	995	Both	
Stonehouse Pizza	FSR	90	Both	
Suburban	FSR	0	2018	No UK website
Subway	FF	2229	Both	
Table Table	FSR	80	Both	
Taco Bell	FF	50	Both	
Taylor Walker	FSR	0	2018	Ceased trading
Tim Hortons	CCB	27	Both	
Toby Carvery	FSR	151	Both	
Two for One	FSR	90	Both	
Vintage Inns	FSR	186	Both	
Wagamama	FSR	149	Both	
Walkabout	FSR	21	2018	No nutrition
Wasabi	QSR	39	2018	No nutrition for desserts
Wetherspoons	FSR	861	Both	
Wild Bean Café	CCB	350	Both	
Wimpy	FF	71	Both	
Yates's	FSR	29	2018	No nutrition for desserts
Yo! Sushi	FSR	80	Both	
Zizzi	FSR	130	(2020)	Excluded after data cleaning
