# Supplementary material for: Tackling (Childhood) Obesity through a Voluntary Food Reformulation Policy: A Repeated Cross-Sectional Study Investigating Nutritional Changes in the Out-of-Home Sector
Source: Nutrients. 2023 Jul 14;15(14):3149. doi: 10.3390/nu15143149 (PMC10384819; doi:10.3390/nu15143149)

Supplementary File S3.

Figure S1. Boxplots showing distributions of (a) sugar, (b) energy, (c) fat and (d) saturated fat per portion of dessert by category and year

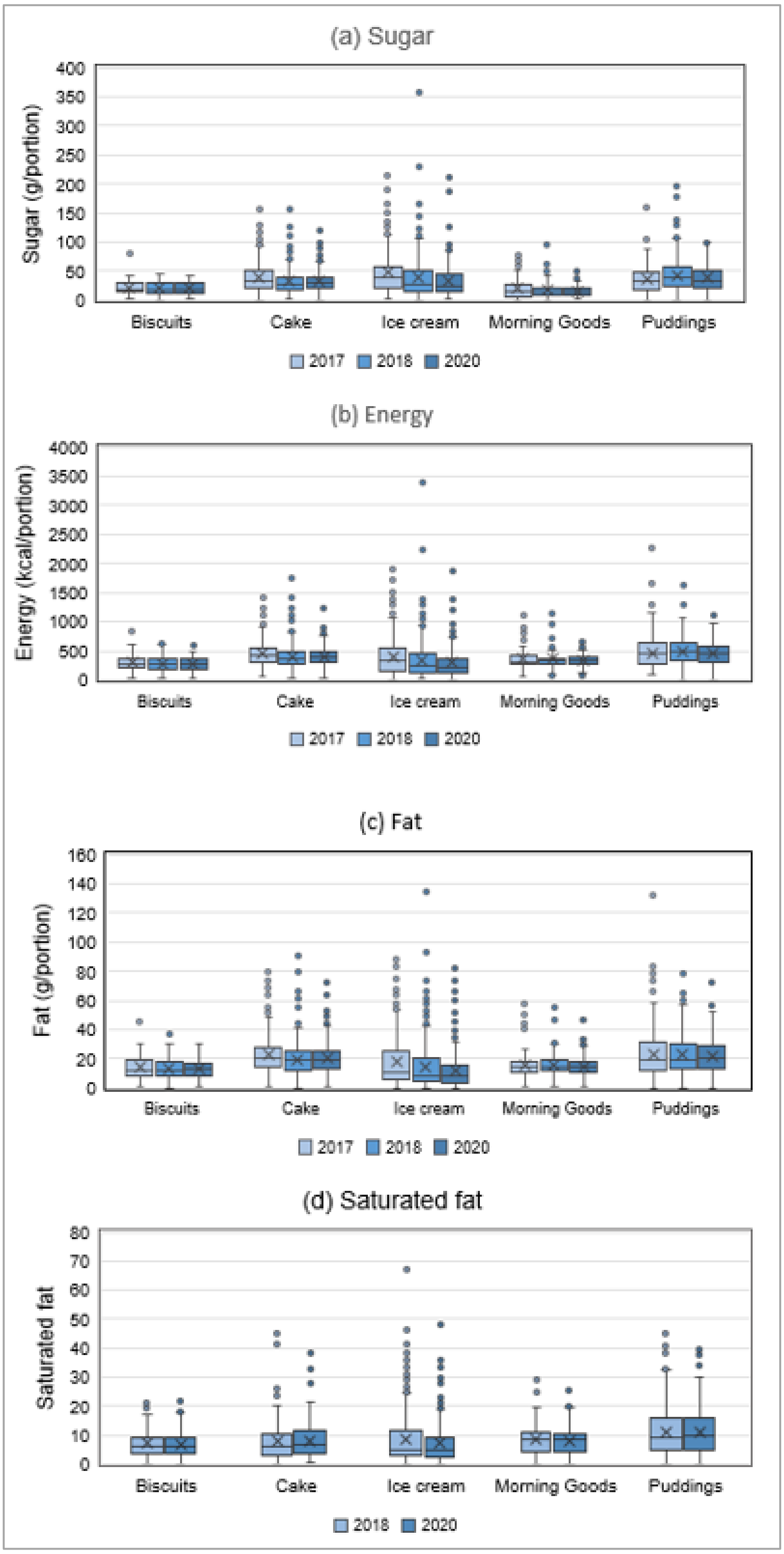

Supplement: Supplementary file 1 [file nutrients-15-03149-s001.zip › Supplementary File S3. Distributions of sugar, energy, fat and saturated fat by category and year.pdf]
