# Supplementary material for: Tackling (Childhood) Obesity through a Voluntary Food Reformulation Policy: A Repeated Cross-Sectional Study Investigating Nutritional Changes in the Out-of-Home Sector
Source: Nutrients. 2023 Jul 14;15(14):3149. doi: 10.3390/nu15143149 (PMC10384819; doi:10.3390/nu15143149)
Supplement: Supplementary file 1 [file nutrients-15-03149-s001.zip › Supplementary File S4. Descriptive statistics for subset with per 100g data.pdf]

Supplementary File S4:

Table S3. Descriptive statistics for subset with per 100g data

**Menu**		**Mean**	**Standard deviation**	**Median**	**Inter-quartile range**
Adult (n=751)	Sugar g/100g	25.3	9.78	25.0	18.9 - 32.0
	Fat g/100g	17.6	7.77	17.4	12.8 - 22.3
	kcal/100g	3.6	1.00	3.7	3.0 - 4.3
	Sugar g/portion	24.0	13.50	22.4	15.0 - 31.2
	Fat g/portion	16.0	8.79	15.4	10.0 - 21.0
	Saturated fat g/portion	7.5	5.20	6.9	3.6-10.2
	Portion weight g	98	50.2	90	67 - 120
	kcal/portion	331	141.2	325	237 - 398
Child (n=50)	Sugar g/100g	22.3	8.81	23.4	17. 0 - 35.4
	Fat g/100g	6.4	5.7	6.5	0.2 - 13.3
	kcal/100g	1.8	1.14	2	0.9 - 3.9
	Sugar g/portion	16.5	8.11	16.3	10.8 - 25.3
	Fat g/portion	4.5	4.18	4.6	0.1 - 9.6
	Saturated fat g/portion	2.7	2.54	2.95	0.1-3.7
	Portion weight g	78	38.0	72	51 - 129
	kcal/portion	130	83.5	122.5	70 - 239
